# Lutein and zeaxanthin isomers modulates lipid metabolism and the inflammatory state of retina in obesity-induced high-fat diet rodent model

**DOI:** 10.1186/s12886-017-0524-1

**Published:** 2017-07-24

**Authors:** Mehmet Tuzcu, Cemal Orhan, Omer Ersin Muz, Nurhan Sahin, Vijaya Juturu, Kazım Sahin

**Affiliations:** 1 Faculty of Science, Division of Biology, Firat University, Elazig, Turkey; 2 Faculty of Veterinary, Department of Animal Nutrition, Firat University, Elazig, Turkey; 3 Department of Ophthalmology, Elazig Education and Research Hospital, Elazig, Turkey; 4 Research and Development, OmniActive Health Technologies Inc., Morristown, USA

**Keywords:** High fat diet, Lutein, Zeaxanthin, NF-κB, Nrf2, Retina

## Abstract

**Background:**

Several studies associated high-fat intakes with a high incidence of age-related macular degeneration (AMD). Lutein and Zeaxanthin isomers (L/Zi) may counteract reactive oxygen species produced by oxidative stress. The present study was conducted to determine the possible effects of L/Zi administration on lipid profile, protein genes associated with oxidative stress and inflammation pathways in the obesity induced by a high-fat diet (HFD) in rodents.

**Methods:**

Twenty-eight male Wistar rats were allocated into four groups as follows: (i) Control, (ii) Control + L/Zi, (iii) High Fat Diet (HFD), and (iv) HFD+ L/Z. L/Zi was administrated for 8 weeks at a daily dose of 100 mg/kg BW.

**Results:**

L/Zi administration significantly reduced insulin and free fatty acid (FFA) levels (*P* < 0.001) and ameliorated the oxidative damage by reducing malondialdehyde (MDA) concentration and increasing antioxidant enzymes activities of retina induced by HFD. In addition, supplementation decreased the levels of vascular endothelial growth factor (VEGF), inducible nitric oxide synthase (iNOS), nuclear factor-kappa B (NF-κB) and intercellular adhesion molecule-1 (ICAM) (*P* < 0.001, respectively) and improved nuclear factor erythroid 2-related factor 2 (Nrf2) and heme oxygenase 1 (HO-1) gene proteins in retinal tissues (*P* < 0.001).

**Conclusion:**

Rats fed with HFD exhibited increased oxidative stress and upregulation of inflammatory indicators. However, L/Zi supplementation modulates genes involved oxidative stress and inflammation including NF-κB and Nrf2 signaling pathways in the retina which may contribute to ameliorating retinal damage induced by HFD.

## Background

Age-related macular degeneration (AMD) is one of the most common causes of visual impairment in the elderly population in developed countries [1, 2]. There are 2 types of the disease, dry and wet type [3]. Dry AMD has a stable course, but wet AMD leads to progressive visual loss by causing hemorrhage, edema and choroidal membrane formation in the fovea. Wet type AMD has no definitive treatment but the most effective treatment option today is intravitreal injections of anti-VEGF drugs [3–5]. Approximately monthly injections were required and in the UK, the total annual cost is about 1 billion euros [6]. Due to the frequent manifestation of the disease, the probability of severe complications such as endophthalmitis related with intravitreal injections, and the high cost, the preventive strategies from the disease have gained importance. Age and obesity correlated with AMD risk [7–13]. The Age-Related Eye Disease Study (AREDS) and AREDS2 were two large randomized clinical trials evaluating the effects of nutritional supplementation on AMD and reported that recommended supplements including vitamin C, vitamin E, lutein (L), zeaxanthin (Z), and zinc might have a positive effect on AMD [14, 15].

Lutein (3R,3’R,6’R-β,ε-caroten-3,3′-diol) and zeaxanthin (3R,3’R-β,β-caroten-3,3′-diol) are the two main xanthophylls which are an oxygenated form of carotenoids (oxycarotenoids). They have the basic C40 isoprenoid structure similar to the other carotenoids and have an ionone ring at each terminal end which contains hydroxyl groups attached to the 3 and 3′ positions [16]. L and Z cannot be synthesized in humans due to the absence of the relevant carotenoid synthesis enzyme. Therefore, dietary intake of carotenoids at an adequate level is important [17]. L and Z exist at higher concentrations in green leafy vegetables and corn products, respectively [18, 19]. These carotenoids contained in the retina, especially at the fovea, much higher concentrations compared to the other tissues. This recommends that L and Z may have a significant role in retinal function and viability [20]. Free oxygen radicals are formed at extreme levels in retina more than other tissues due to the structural properties of the retina including high oxygen concentration due to the excessive blood supply, high amount of polyunsaturated fatty acids and over-exposure of visible light. This disproportionate burden of oxidative stress contributes to the development of many retinal diseases. In several studies, the anti-oxidative properties of L and Z were shown [16, 17]. In addition to anti-oxidative effects, macular pigments filter blue light and regulate photo stress recovery time, macular function, and neural processing speed [17, 21]. Despite known property of macular pigments as an antioxidant, its underlying molecular mechanisms of action remain poorly understood. In the current study, we aimed to investigate the possible effects of L and Z isomers (L/Zi) on lipid profile, protein genes involved oxidative stress and inflammation pathways of the retina in obese rodents induced by a high-fat diet.

## Methods

### Animals and diets

Eight-week-old, 180 ± 20 g weighing, twenty-eight male Sprague-Dawley rats obtained from Laboratory Animal Research Center of Firat University (Elazig, Turkey). Rats were maintained in stable temperature (22 ± 2 °C), humidity (55 ± 5%) and controlled illumination (12/12 h light/dark cycle) and under non-pathogenic conditions. All animal procedures were accepted by the Ethical Committee of Animal Experiments at University of Firat (Elazığ, Turkey). Oxidative damage stress was shown in rats fed with HFD as the study reported by our group [22].

After acclimation for 2 weeks, 28 rats were randomly assigned to four groups as 7 animals in each group as follows: (i) Control, (ii) L/Zi (100 mg L/Zi /kg BW), (iii) High Fat Diet (442% of calories as fat, HFD), (iv) HFD+ L/Zi (100 L/Zi mg/kg BW). L/Zi was administered once a day for 8 weeks. The detailed composition of the regular and high-fat diet is shown in detail in Table 1. The product of L/Zi (Lutemax 2020™) extracted from Marigold flowers (*T. erecta* L) was provided by OmniActive Health Technologies Ltd. (Pune, India). Marigold is a red-orange crystal powder that is a characteristic odor of marigold flower. It is obtained by saponification of and thermal isomerization reaction of an extract comprising a xanthophyll extract such as marigold flower oleoresin. The isomerization reaction transforms certain of the free L to (3R, 3′S) - Z (meso-isomer), while saponification causes the release of free calcium formats in free form (lutein/zeaxanthin). Then, the mix is exposed to extraction, purification and drying under vacuum to obtain the L/Zi product. The product comprises 80% carotenoids with 67% L and 13.5% Z isomers. The isomeric distribution of zeaxanthin in the product is about 50:50 as a mixture of (3R,3′R)-β,β-carotene-3,3′-diol and (3R,3′S)-β,β-carotene-3,3′-diol, usually denoted to as zeaxanthin and meso-zeaxanthin, respectively. Additionally, the L/Zi also includes candles ranging from 14 to 16%. A dosage of 100 mg/kg was selected since many in vivo studies showed that this dosage was antioxidant in rodents [23–25].

**Table 1:** Composition of diets (g/kg diet) fed to rats

	Regular Diet	HFD
Casein	200.0	200.0
Starch	579.5	150.0
Sucrose	50.0	149.5
Soybean oil	70.0	-
Beef tallow	-	400.0
Cellulose	50.0	50.0
Vitamin-Mineral Premix^a^	45.0	45.0
l-cysteine	3.0	3.0
Choline Bitartrate	2.5	2.5

^a^The vitamin-mineral premix provides the following (per kg): all-*trans*-retinyl acetate, 1.8 mg; cholecalciferol, 0.025 mg; all-*rac*-a-tocopherol acetate, 12,5 mg; menadione (menadione sodium bisulfate), 1.1 mg; riboflavin, 4.4 mg; thiamine (thiamine mononitrate), 1.1 mg; vitamin B-6, 2.2 mg; niacin, 35 mg; Ca-pantothenate, 10 mg; vitamin B-12, 0.02 mg; folic acid, 0.55 mg; *d*-biotin, 0.1 mg. manganese (from manganese oxide), 40 mg; iron (from iron sulfate), 12.5 mg; zinc (from zinc oxide), 25 mg; copper (from copper sulfate), 3.5 mg; iodine (from potassium iodide), 0.3 mg; selenium (from sodium selenite), 0.15 mg; choline chloride, 175 mg

At the end of the experimental phase of the study, animals were sacrificed by cervical dislocation. Samples of blood were collected for biochemical analysis. In addition, retinas were removed for biochemistry and Western blot analyses.

### Biochemical and antioxidant enzymes analyses

Blood samples were centrifuged at 3000 *g* for 10 min and then serum glucose, cholesterol, triglyceride and free fatty acids (FFA) levels were determined by a biochemical analyzer (Samsung LABGEO PT10, Samsung Electronics Co., Suwon, Korea). Serum insulin was analyzed using an ultrasensitive rat insulin kit (Linco Research Inc., St. Charles, MO, USA) by an Enzyme-Linked Immunosorbent Assay (ELISA, Elx-800; Bio-Tek Instruments Inc., Vermont, USA). The sensitivity of the assays was 0.28 ng/mL and the interassay and intraassay coefficients of variation were 4.5% and 6.8% for insulin.

Retinal malondialdehyde (MDA) concentration was determined using High-Performance Liquid Chromatography (Shimadzu, Tokyo, Japan) by a UV-vis SPD-10 AVP detector and 30 mM KH2PO4 and a CTO-10 AS VP column at a flow rate of 1.2 ml/min [26]. Column waste was monitored at 250 nm and the volume was 20 μl. Retinal homogenate (10%, w / v) was prepared in 10 mM phosphate buffer (pH 7.4), centrifuged at 13,000 x g for 10 min at 4 °C. Total antioxidant capacity (TAC) was determined by Erel’s method [27] as previously described, based on the bleaching of the characteristic color of a more stable blue-green 2,2′-azino-bis 3-ethylbenzothiazoline-6-sulfonate (ABTS) by antioxidants. The results were given in mmol Trolox equivalents / L.

Antioxidant enzymes (SOD, CAT, and GSH-Px) activities in the homogenate were determined according to the supplied with the commercial kit (Cayman Chemical, Ann Arbor, MI, USA).

### Western blot

Fresh retina samples were quickly collected for homogenization after scarification. Then samples homogenized in phosphate buffered saline (PBS) including protease inhibitor cocktail and subsequently total protein level was determined. 50 μg per homogenate was stirred with sample buffer and after 5 min of boiling samples were separated by SDS-PAGE and electroblotted on nitrocellulose membranes. The membranes were washed in PBS and blocked by a 1% bovine serum albumin (BSA) for 1 h before the administration of primary antibody. Nitrocellulose membranes were incubated overnight at 4 °C with primary antibodies against vascular endothelial growth factor (VEGF), inducible nitric oxide synthase (iNOS), intercellular adhesion molecule1 (ICAM1), nuclear factor kappa B (NFκB), nuclear factor erythroid 2 related factor 2 (Nrf2), heme oxygenase 1 (HO1) and ß-actin [22]. All the primary and secondary antibodies were purchased from Abcam (Abcam, Cambridge, UK). Secondary antibodies against VEGF, iNOS, ICAM, NFκB, Nrf2, and HO1 were diluted at a concentration of 1:1000 in the same buffer including 0.05% Tween 20. Densitometric analyses of the bands were performed with software (Image J, National Institute of Health, Bethesda, USA).

### Statistical analysis

The size of the sample was calculated based on a *P*-value of 0.05 and a magnitude of 85%. All analyses were performed using the General Linear Model procedure (SAS, 2002). Differences among groups were analyzed by Fisher’s post hoc test.

## Results

### Changes in metabolic health and lipid profile

**Fig. 1:**
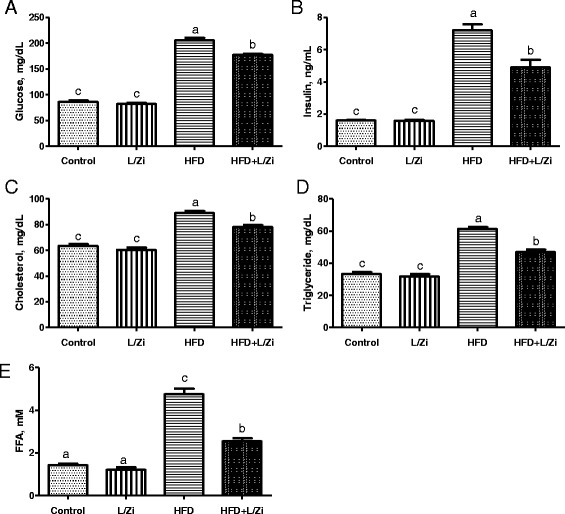
The effects of lutein and zeaxanthin isomers on the concentrations of glucose (Panel **A**) insulin (Panel **B**) cholesterol (Panel **C**) tryglyceride (Panel **D**) and free fatty acid FFA, (Panel **E**). Values are expressed as the mean ± standard error (*n*=7 per group). FFA, free fatty acid; L/Zi, control + lutein and zeaxanthin isomers; HFD, high-fat diet; HFD+L/Zi, high-fat diet + lutein and zeaxanthin isomers

Following the feeding with HFD, abnormal levels of the glucose, insulin, cholesterol, triglyceride, and FFA appeared in the serum of rats (Fig. 1A-E). When compared with the control group, there was no significant difference in the releases glucose, insulin, cholesterol, triglyceride and FFA in group lutein and zeaxanthin (*P* > 0.05; Fig. 1). Nevertheless, L/Zi supplementation for 8 weeks was attenuated by reduction of glucose, insulin, cholesterol, triglyceride and FFA compared to group HD (*P* < 0.001).

### Changes in MDA levels and antioxidant enzymes

**Fig. 2:**
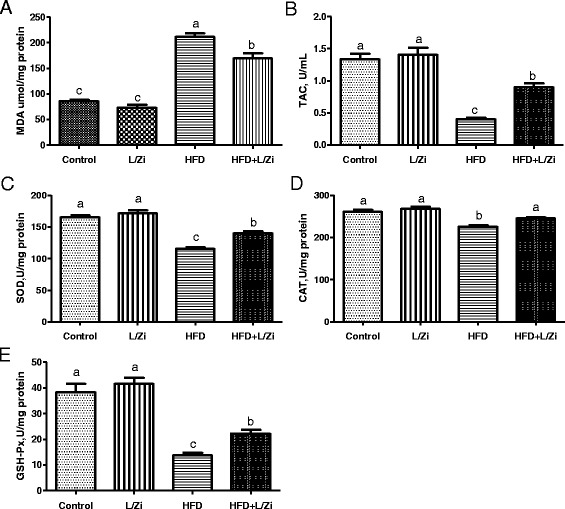
The effects of lutein and zeaxanthin isomers on retina antioxidant status. Values are expressed as the mean ± standard deviation (*n* = 7 per group). MDA, malondialdehyde; TAC, total antioxidant capacity; SOD, superoxide dismutase; CAT, catalase; GSH-Px, glutathione peroxidase; L/Zi, control + lutein, and zeaxanthin isomers; HFD, high-fat diet; HFD + L/Zi, high-fat diet + lutein and zeaxanthin isomers

The retinal oxidative status was investigated by identifying markers for oxidative stress including MDA; markers for anti-oxidative defense system including TAC, and three important antioxidant enzyme activities, GSH-Px, CAT, and SOD. As shown in Fig. 2 panel A-E, HFD induced an excessive release of MDA, a decrease of TAC levels and reduced the SOD, CAT and GSH-Px activity in the retinal tissue of HFD-fed rats compared to group control (*P* < 0.001). L/Zi significantly reduced MDA levels and improved TAC levels and SOD, CAT and GSH-Px activity in the retina of HFD-fed rats (*P* < 0.001). The results demonstrate that HFD causes oxidative stress by increasing the levels of oxidation products and weakening the activity of antioxidant enzymes and can be reversed with L/Zi supplementation.

### Change in retinal VEGF, iNOS, ICAM-1, NF-κB, Nrf-2, and HO-1 protein levels

**Fig. 3:**
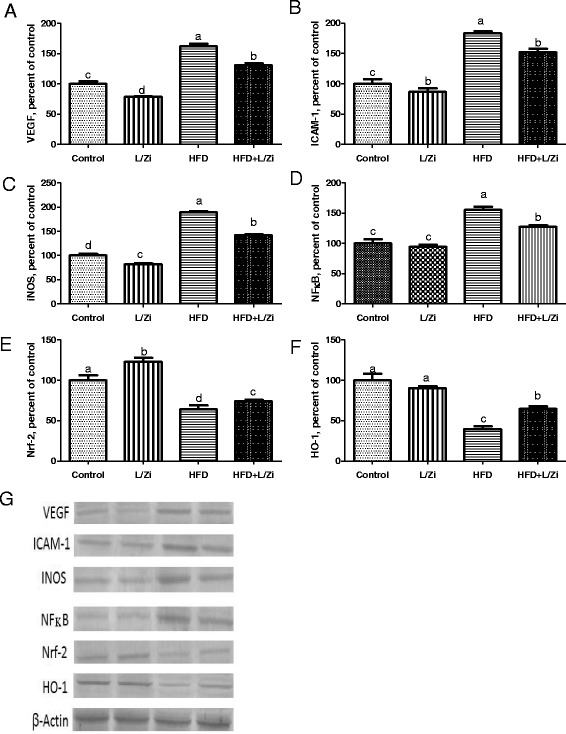
The effects of lutein and zeaxanthin isomers on VEGF (Panel **A**), ICAM-1 (Panel **B**), INOS (Panel **C**), NFkB (Panel **D**), Nrf-2 (Panel **E**) and HO-1 (Panel **F**) levels of retina tissue in rats. The intensity of the bands shown in (Panel **G**) was quantified by densitometric analysis. VEGF, vascular endothelial growth factor; ICAM-1, intercellular adhesion molecule 1; iNOS, inducible nitric oxide synthase; NF-kB, NF-kappa-B transcription complex; Nrf-2, nuclear factor erythroid 2 [NF-E2]-related factor 2; HO-1, heme oxygenase-1; L/Zi, control + lutein and zeaxanthin isomers; HFD, high-fat diet; HFD+L/Zi, high-fat diet+lutein and zeaxanthin isomers. The intensity of the bands was quantified by densitometric analysis. Data are expressed as a ratio of normal control value (set to 100%). The bar represents the standard error of the mean. Blots were repeated at least 3 times (*n*=3) and a representative blot is shown. β-actin was included to ensure equal protein loading

As shown in Fig. 3 (Panel A-F), VEGF, ICAM-1, iNOS and NF-κB levels increased and a decrease of Nrf-2 and HO-1 levels in the retina was observed in HFD rats (*P* < 0.001). HFD + L/Zi supplementation decreased VEGF, ICAM-1, iNOS and NF-κB expression (*P* < 0.001). Compared with HFD group, Nrf-2, and HO-1 levels were increased in rats treated with HFD + L/Zi (*P* < 0.001). These results suggest that L/Zi supplementation may regulate antioxidant signaling pathways to protect cells from oxidative stress.

## Discussion

Findings from various studies suggest that supplementation of L and Z alone support the protective and therapeutic effect of various eye diseases such as age-related macular degeneration, diabetic retinopathy, cataract and ischemic/hypoxia-related retinopathy, and slight retinal damage, retinitis pigmentosa, retinal detachment and uveitis [25, 28, 29]. The present study has demonstrated the following main findings: HFD intake was related to increased serum glucose, insulin, cholesterol, triglyceride and FFA levels in rats while L/Zi decreased metabolic and lipid profile risk markers. L/Zi also regulates HFD-induced oxidative stress by increasing the activity of SOD, CAT, GSH-Px, and TAC and reducing MDA in the retinal tissue of rats. Supplementation of L/Zi markedly ameliorated the increased expression of VEGF, iNOS, ICAM1, and NFκB and decreased expression Nrf2 and HO1 by HFD in retinal tissue in animals.

The pathogenesis of retinopathy is extremely likely to be facilitated by inflammatory progressions including leukocyte adhesion and the cytokine system [30, 31]. High-fat diet-induced hyperglycemia is a factor which is recognized to alleviate the level of VEGF and ICAM-1 [22]. VEGF, a strong angiogenic and proinflammatory factor, is raised in the retina and vitreous animals fed with HFD and diabetic rats, and are associated with the appearance of this retinopathy [22, 32]. In an in vitro study, it was found that VEGF level in Muller cells may contribute to imbalance under high glucose concentration, an imbalance between angiogenic stimulants and inhibitors, retinal neovascularization in diabetic retinopathy [32]. In addition, retinopathy has been revealed to be an inflammatory factor and leukostasis and increased levels of ICAM1, a member of the 80–114 kD immunoglobulin gene superfamily, which has been observed in retina in diabetes and hyperglycaemia [22, 33]. However, the relations between VEGF, ICAM1 and L/Zi under high hyperglycemia remain unclear. In this study, HFD rats had higher VEGF, ICAM1 and iNOS levels in the retina than control rats (Fig. 3) and regulated with L/Zi. Because of the lack of prior literature, examining the properties of L/Zi supplementation on these protein expression in the retina of HFD rats, the present data cannot be compared with the literature. Therefore, the beneficial effect of L/Zi support on reduced VEGF, iNOS and ICAM1 levels is important and L/Zi has a possible role in preventing the progress of retinal impairment. Many studies have reported that antioxidants that act as lutein and zeaxanthin inhibit elevated VEGF levels in retinas of diabetic rats [34, 35]. Fernández-Robredo et al. [36] reported that apolipoprotein E- deficiency (ApoE−/−) caused an elevation of VEGF, vacuole formation in retina pigment epithelium (RPE), deposit accumulation in basal lamina and an increase in the thickness of Bruch’s membrane similar to the findings observed in AMD. However, supplementation of Z, ascorbate, tocopherol, and zinc to diet improved retinal alterations and reduced VEGF expression in the retina of ApoE−/− mice [36]. Multivitamin plus L and glutathione complex compared to L alone were found to be more successful in ameliorating retinal changes and reducing VEGF expression and MMP-2 activity in ApoE−/− mice model [37]. A mice model [DKO; Chemokine (C-C motif) ligand 2 (Ccl2(−/−))/CX3C chemokine receptor 1 (Cx3cr1(−/−) mice on Crumbs homolog 1 retinal degeneration phenotype 8 (Crb1(rd8) background] showed a more retinal expression of tumor necrosis factor-alpha (TNF-*α*), cyclooxygenase-2, interleukin-1 beta (IL-1*β*), iNOS and VEGF and developed focal retinal lesions including photoreceptor and RPE degeneration [38]. Similarly, Ramkumar et al. [38] demonstrated that the AREDS2 formulation (L, Z, long-chain n3 polyunsaturated fatty acid, docosahexaenoic acid and eicosapentaenoic acid) ameliorated pathological findings and decreased overexpression of inflammatory and angiogenic genes in DKO mice. Additionally, increasing in MDA and NF-κB levels and decreasing in glutathione and GSH-Px activities of the retina were observed with L supplementation in alloxan-induced diabetic mice under hyperglycemic conditions [39]. In another study, Kowluru et al. [35] reported that retinal lipid peroxidation, oxidative modifying DNA, nitrotyrosine, iNOS, VEGF and ICAM-1 were elevated and the expression of electron transport complex III, Mn superoxide dismutase and GSH was decreased in diabetic rats. However, Z (0.02% and 0.1%) ameliorated these parameters except for GSH. Both 0.02% and 0.1% Z doses have similar effects on diabetic-induced retinal abnormalities. With the supplementation of L, Z, and multi-nutrition, improvement in pathological findings (increased capillary cell apoptosis and vascular pathology), inhibition of NF-κB and decrease in the VEGF and IL-1*β* expression were reported [35]. However, antioxidant and anti-inflammatory effects of nutrition were not accompanied by the control of hyperglycemia. These results suggest the beneficial properties of macular carotenoid containing nutritional supplements on diabetes-induced retinal pathology were independent of hyperglycemia control [40].

Oxidative stress caused by hyperglycemia plays an important role in inflammatory gene expression through the activation of transcription factors. One of them, NF-κB is a widely transcribed factor that is over-exaggerated everywhere and controls several of genes associated with inflammatory and immune responses [41]. Under physiological circumstances, the production and removal of reactive oxygen species is completely regulated and does not create redundant inflammation in the body. But, continuous oxidative stress resulting from HFD and diabetes is the main reason of retinal inflammation. NF-κB activity is increased in retinal endothelial cells treated with high glucose, pericytes, or glial cells, and in in vivo studies [42–44]. Another transcription factor, Nrf2 shows a key role in the initiation of phase II detoxifying/antioxidant protection mechanisms to deal with oxidative stress by increasing the expression of a number of enzymes such as NAD (P) H-quinone oxidoreductase 1, glutamate-cysteine-1, glutathione S-transferase and UDP-glucuronosyltransferase [45]. Carotenoids can up-regulate the antioxidant electrophile / antioxidant response element (EpRE/ARE) and interact with Nrf2 to block oxidative stress and motivate phase II enzymes and protect antioxidants such as glutathione-S-transferases from reactive oxygen species and other electrophilic molecules [22, 46, 47]. The studies on NFκB and Nrf2/HO1 signaling pathway of L/Zi supplementation in HFD rats are limited. In this study, it was found that increased NFκB activity and decreased Nrf2 and HO1 levels in HFD rats were associated with activation and translocation, respectively, to derive from HFD-induced oxidative stress (Fig. 3). Due to the literature limitation, the properties of L/Zi supplementation on the activation of NFκB and Nrf2 / HO1 in the retinas of HFD rats are not comparable. Nevertheless, it has earlier been reported that carotenoids inhibited NFκB expression and increased Nrf2 level in cisplatin-induced nephrotoxic kidneys in rats [48]. Following induction of uveitis in rats by subcutaneous injection of lipopolysaccharide, various inflammatory factors significantly increased in the aqueous humor and ocular tissues. Jin et al. [49] reported an increase in levels of TNF-α, interleukin-6 (IL-6), monocyte chemoattractant protein-1, macrophage inflammatory protein-2, nitric oxide (NO), activation of NFκB in the iris-ciliary body. However, intravenous L injection at a dose of 100 mg/kg enhanced all results, including inhibition of the NFκB pathway and subsequent production of pro-inflammatory mediators. In the endotoxin-induced uveitis animal model, NO and lipid peroxidation concentrations were increased while oxygen radical absorbance capacity, total SOD, GSH and GSH-Px activities and expression of mRNA copper-zinc SOD, manganese SOD, and GSH-Px were reduced in the ocular tissues [50]. But, above-mentioned changes related to oxidative stress were reversed by supplementation of oral L. In addition, it was suggested that Z might activate the Nrf2 pathway [51]. Taken together, our results suggest that L/Zi could be an appropriate co-adjuvant treatment for retinal changes in rats fed with HFD via ameliorating oxidative stress and inhibition of VEGF and NFκB pathway and inflammation and activation of Nrf2 pathways.

## Conclusion

The results of this study suggest that HFD may disrupt metabolic profile and lead to oxidative damage. In addition, inflammation cascade can be triggered by increased VEGF, I-NOS, ICAM-1 and NF-κB levels and reduced Nrf2 and HO-1 levels. These changes in the retina can lead to AMD-like retinopathy and could be partially reversed by the supplemental L and Zi.

## Abbreviations

ABTS: 2,2′-azino-bis 3-ethylbenzothiazoline-6-sulfonate
AMD: Age-related macular degeneration
ApoE−/−: Apolipoprotein E- deficiency
AREDS: The Age-Related Eye Disease Study
BW: Body weight
CAT: Catalase
COX-2: Cyclooxygenase-2
DKO: Chemokine (C-C motif) ligand 2 (Ccl2(−/−))/CX3C chemokine receptor 1 (Cx3cr1(−/−)) mice on Crumbs homolog 1 retinal degeneration phenotype 8 (Crb1(rd8)) background
FFA: Free fatty acid
GSH: Glutathione
GSH-Px: Glutathione peroxidase
HEPES: 2-[4-(2-Hydroxyethyl)-1-piperazinyl]ethanesulfonic acid
HFD: High-fat diet
HO-1: Heme oxygenase 1
ICAM: Intercellular adhesion molecule-1
IL-1*β*: Interleukin-1 beta
IL-6: Interleukin-6
iNOS: Inducible nitric oxide synthase
I-κB: Inhibitor kappaB
L: Lutein
L/Zi: Lutein and Zeaxanthin isomers
MDA: Malondialdehyde
NF-κB: Nuclear factor-kappa B
NO: Nitric oxide
Nrf2: Nuclear factor erythroid 2-related factor 2
PMSF: Phenylmethylsulfonyl-fluoride
RPE: Retina pigment epithelium
SOD: Superoxide dismutase
TAC: Total antioxidant capacity
TNF-*α*: Tumor necrosis factor-alpha
VEGF: Vascular endothelial growth factor
Z: Zeaxanthin
